# Does a Mobile Phone Depression-Screening App Motivate Mobile Phone Users With High Depressive Symptoms to Seek a Health Care Professional’s Help?

**DOI:** 10.2196/jmir.5726

**Published:** 2016-06-27

**Authors:** Nasser F BinDhim, Eman M Alanazi, Hisham Aljadhey, Mada H Basyouni, Stefan R Kowalski, Lisa G Pont, Ahmed M Shaman, Lyndal Trevena, Tariq M Alhawassi

**Affiliations:** ^1^ College of Health Sciences Health Informatics Saudi Electronic University Riyadh Saudi Arabia; ^2^ College of Pharmacy King Saud University Riyadh Saudi Arabia; ^3^ Medication Safety Research Chair King Saud University Riyadh Saudi Arabia; ^4^ The Smart Health Project Riyadh Saudi Arabia; ^5^ School of Pharmacy and Medical Sciences University of South Australia Adelaide Australia; ^6^ Sansom Institute for Health Research University of South Australia Adelaide Australia; ^7^ Centre for Health Systems and Safety Research Australian Institute of Health Innovation Macquarie University Sydney Australia; ^8^ Public Health School University of Sydney Sydney Australia

**Keywords:** mental health, depression, mobile phone, public health informatics, patients’ screening

## Abstract

**Background:**

The objective of disease screening is to encourage high-risk subjects to seek health care diagnosis and treatment. Mobile phone apps can effectively screen mental health conditions, including depression. However, it is not known how effective such screening methods are in motivating users to discuss the obtained results of such apps with health care professionals. Does a mobile phone depression-screening app motivate users with high depressive symptoms to seek health care professional advice? This study aimed to address this question.

**Method:**

This was a single-cohort, prospective, observational study of a free mobile phone depression app developed in English and released on Apple’s App Store. Apple App Store users (aged 18 or above) in 5 countries, that is, Australia, Canada, New Zealand (NZ), the United Kingdom (UK), and the United States (US), were recruited directly via the app’s download page. The participants then completed the Patient Health Questionnaire (PHQ-9), and their depression screening score was displayed to them. If their score was 11 or above and they had never been diagnosed with depression before, they were advised to take their results to their health care professional. They were to follow up after 1 month.

**Results:**

A group of 2538 participants from the 5 countries completed PHQ-9 depression screening with the app. Of them, 322 participants were found to have high depressive symptoms and had never been diagnosed with depression, and received advice to discuss their results with health care professionals. About 74% of those completed the follow-up; approximately 38% of these self-reported consulting their health care professionals about their depression score. Only positive attitude toward depression as a real disease was associated with increased follow-up response rate (odds ratio (OR) 3.2, CI 1.38-8.29).

**Conclusions:**

A mobile phone depression-screening app motivated some users to seek a depression diagnosis. However, further study should investigate how other app users use the screening results provided by such apps.

## Introduction

Great computational and storage abilities as well as proximity to users potentially make mobile phone apps excellent health research tools that are capable of delivering complex health interventions [1]. In addition, research participants can be recruited directly from app stores [2,3]. Hence, various studies have explored the use of mobile phone apps locally and cross-country for health research, including cross-sectional studies [2,3], observational studies [4], and randomized controlled trials [5,6].

### Mobile phone and Mental Health Screening

Current research on apps for screening and monitoring mental health has shown feasibility across diverse ranges of mental health conditions, including depression [3], bipolar disorder [7,8], anxiety disorders [9,10], and substance abuse disorders [2,5,6-11]. Furthermore, recent studies suggest that mobile phone ownership is very common among mental health patients and they have a strong interest in using mobile phones to monitor their mental health [12,13]. However, there is still very limited evidence regarding the efficacy of mobile phone-delivered mental health interventions or screening tools [14]. The limited quantity of studies conducted in this domain might be due to the lack of feasibility and low confidence that these interventions and/or screening tools will reach the targeted populations [3], and may be related to the fact that mobile phone technology is still nascent compared with other delivery channels for health interventions.

In a recent cross-sectional study, 8241 users from 66 countries from Apple’s App Store downloaded a depression-screening app [3]. A high percentage (73.9%) of app downloaders also submitted responses to the screening questionnaire [3], with 25.7% reporting that they had previously been diagnosed with depression [3]. Using two cutoff thresholds of the Patient Health Questionnaire (PHQ-9) depression screening tool, it was found that a large number of participants had high depressive symptoms yet were undiagnosed [3-15]. The studied app also reached various groups of people who were not previously diagnosed with depression yet had high depressive symptoms, and the app was able to reach a group of participants at risk of suicide [3]. In another study in South Korea, 27,159 participants were screened for bipolar spectrum disorders within a few months [8].

### Mobile Phone Apps’ Potential in Mental Health Screening and Monitoring

Active data, in the form of questionnaires for mental health screening or monitoring, can bring clinical assessments from outside of the health care setting into the real-life environment, lived and experienced by patients [16]. In addition, there are dozens of mobile phone apps that provide mental health and depression screening, but few advise users to discuss results with their health care professionals and most do not provide any guidance about how to use such results. Due to the wide popularity of screening tests on mobile phone apps and on the Internet, people are likely to access and use apps, especially if aware of their depressive symptoms. However, once they are aware of the potential seriousness of symptoms, it is critical they take appropriate action and seek professional follow-up by discussing the results with a health care professional. However, it is not yet known how effective such screening methods are in motivating users to seek appropriate professional help. As the objective of screening for disease is to discover the undiagnosed problem so that the users can be placed under treatment [17], establishing an association between mobile phone self-screening for mental health conditions and the actions taken by the users based on their screening results is a critical step in assessing the feasibility, efficacy, and cost-effectiveness of such screening and monitoring methods.

The aim of this study was to address the following question: Does a mobile phone depression-screening app motivate users with high depressive symptoms to seek health care professional advice?

## Methods

### Design

This study was a single-cohort, prospective, observational study of a free mobile phone depression app that was developed in English utilizing the “Health Monitor” app template [18] and was released on Apple’s App Store. The users of the Apple’s App Store from any country can download the app after consenting to provided participant information; however, we limited the app availability to 5 countries: Australia, Canada, New Zealand (NZ), the United Kingdom (UK), and the United States (US). We selected these countries based on their high download and response rates in a previous feasibility study [3]. Users from other countries were excluded because, as provided by a previous study, the app stores function of limiting app users to specific countries in not fully accurate [2]. The research ethics committee at King Saud University approved this study.

### Participants

**Figure 1:**
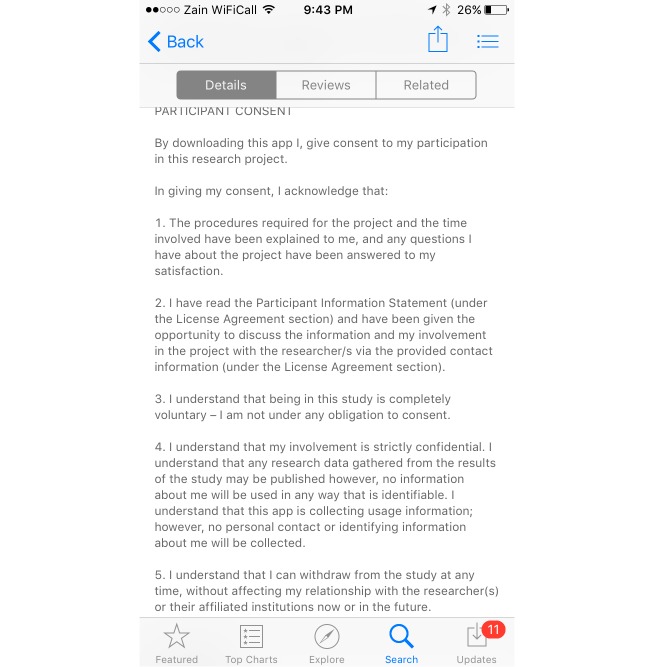
Screenshot of the app download page.

Apple App Store users aged 18 or above, from the 5 nominated countries were recruited directly via the app’s download page in the Apple App Store. The consent and participants’ information are summarized in the app download page (Figure 1) and are included in the “about” section of the app. The studied app was published during the recruitment period (January 25 to March 25, 2015) to the Apple App Store, which was the main portal for advertising this study’s app. In addition, to boost the recruitment process, we advertised the app with demographic targeting (by country) using an in-app advertisement, that results in the app ad being displayed to Apple iPhone users while they are using other apps.

### Recruitment and Data Collection

**Figure 2:**
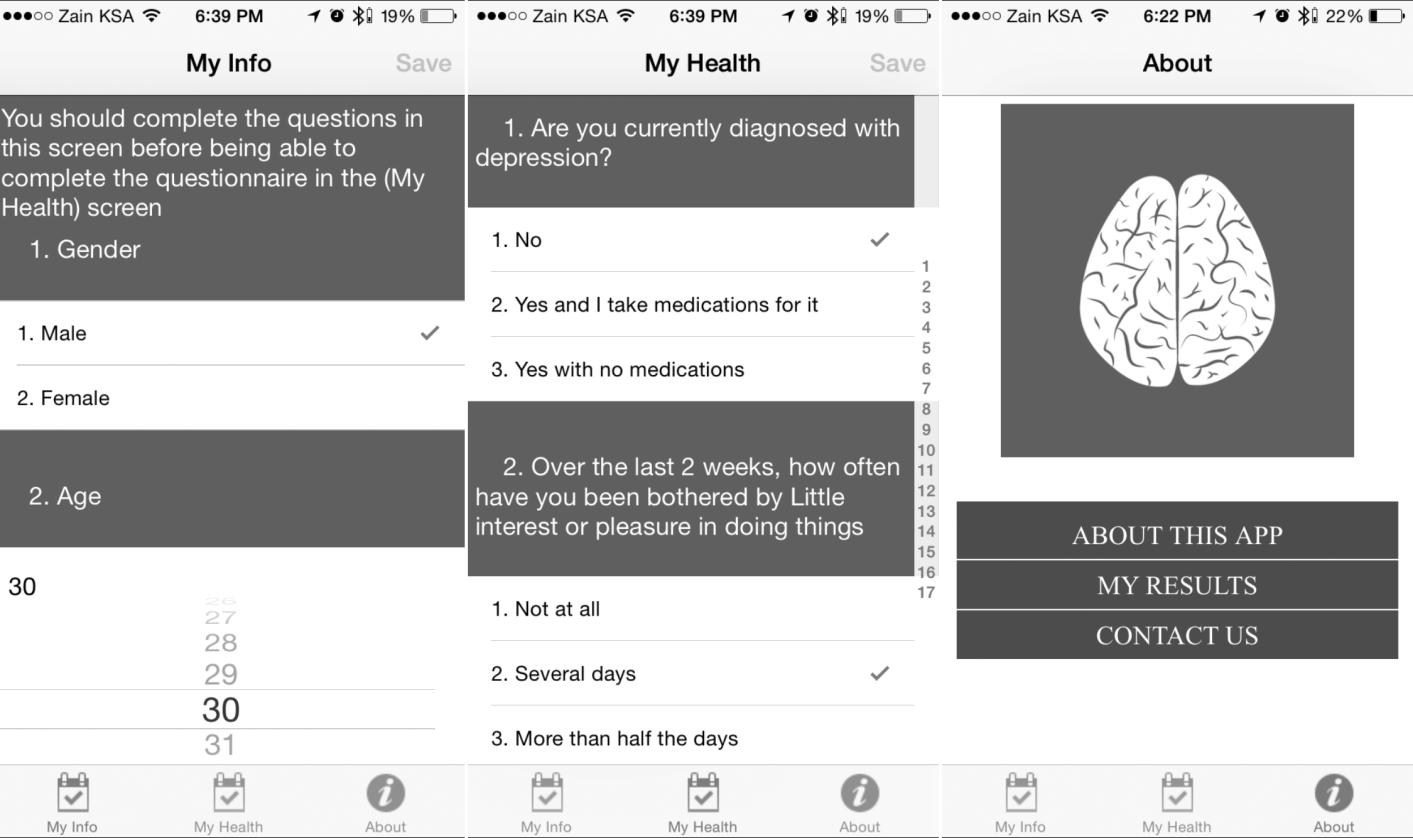
Screenshots of the app.

When a participant downloaded the app and ran it for the first time, the app assigned a unique device identifier to the user’s device and registered it in our secure online research database. The identifier will not change even if the user deletes the app or resets the device. This allowed anonymous data collection and prevented duplicate enrollments. After submitting demographic and baseline data on the first screen of the app, which included educational level, employment and income status, and other health-related characteristics such as chronic conditions, (Figure 2) the app users were able to complete the PHQ-9 on the second screen, including previous depression diagnosis and treatment as well as other depression risk factors, and were able to see their depression screening score. The PHQ-9 was selected over other depression screening tools because (1) it has been validated for use among various age groups [19-21] and (2) there appears to be strong correlation between scores reported from the app and scores reported on paper, with app-collected scores 3.02 (SD 2.25) points higher on average [22].

If the users get a score of 11 or more, the app recommends they discuss the test score with a health care professional. A threshold score of 11 or above was selected based on the literature, which, in pooled estimates of 10 studies had the best trade-off between sensitivity, 0.89 (95% CI 0.75 to 0.96), and specificity, 0.89 (95% CI 0.79 to 0.94) [23]. It is important to note that our app did not provide a diagnosis to the user as our main intention was to replicate the available mental health screening tests on the mobile phone app stores or the Internet. The classification of participants based on the PHQ-9 cutoff 11 was used internally and was not communicated to the participants.

In addition, all of the participants with PHQ-9 scores of 11 or above who did not indicate a previous diagnosis of depression were listed for automated follow-up after 1 month using push notifications. Push notification permits the database server to send messages to a specific user (similar to mobile short message service) at specified times or after a specific task, free of cost [24]. The 1-month follow-up period was selected arbitrarily because there are no specific guidelines regarding a definitive follow-up period but it struck a balance between clinical responsibility and effective push notification practicality. However, at the follow-up, we asked participants if they had discussed the results the app provided with a health care professional, and if so, that professional had diagnosed them with depression or not. We sent 5 push notification reminders over a 10-day period to help increase the follow-up response rate.

### Data Analysis

The mean and SD of the quantitative variables were presented if they had a normal distribution, or the median and range were presented, as appropriate, and compared using *t* tests. We presented the categorical variables as percentages and CIs, and compared using Pearson’s chi-square test. As this study used automated electronic data collection, there were no missing values in the baseline data; the app also includes a data integrity check to prevent users from entering invalid data (such as a maximum age of 99). We used logistic regression to explore factors associated with response rate to the follow-up and with seeking professional help.

## Results

### Uptake and Demographics

In 2 months, 3984 users downloaded the app and 2689 out of 3984 (67.49%) completed the PHQ-9 screening. Of those who completed the screening, 151 users were not eligible for follow-up because they were not from the 5 countries targeted in this study and we were unable to identify from which country were. Thus, 2538 participants were included in this study and were eligible for follow-up. Table 1 shows the participants’ demographics.

**Table 1:** Demographics.

Characteristics		Total n (%)
Age (median range) (years)		27.0 (18-75)
Sex		
	Female	1143 (45.0)
	Male	1395 (54.9)
Country		
	Australia	283 (11.2)
	Canada	541 (21.3)
	United Kingdom	1108 (43.7)
	United States	497 (19.6)
	New Zealand	109 (4.3)
Education		
	Above high school	1177 (46.4)
	High school or less	1361 (53.6)
Income level		
	Less than US $20,000/year	1276 (50.3)
	US $21,000-49,000/year	720 (28.4)
	More than US $50,000/year	542 (21.4)
Employment status		
	Employed	1151 (45.4)
	Self-employed	285 (11.2)
	Student	593 (23.4)
	Unemployed	509 (20.0)

**Table 2:** Country-based prevalence of undiagnosed higher risk of depression using the PHQ-9 threshold of 11.

	Country					
	Australia	Canada	New Zealand	United Kingdom	United States	Total
Low depressive symptoms (PHQ 11)	206 (67.3%)	390 (71.8%)	66 (72.5%)	774 (69.9%)	368 (74.0%)	1804 (70.9%)
High depressive symptoms (PHQ 11)	100 (32.7%)	153 (28.2%)	25 (27.5%)	334 (30.1%)	129 (26.0%)	741 (29.1%)

### Depression Screening and Follow-Up

There were 741 participants out of 2538 (29.1%) with high depressive symptoms (PHQ ≥ 11) as shown in Table 2, of which 419 (56.5%) had been previously diagnosed with depression.

A total of 322 participants, therefore, fulfilled the study criteria for follow-up reminder messages. Of those who followed up, 239 out of 322 (74.2%) completed the follow-up questions. Addressing the principal aim of this study, 91 out of 239 (38%) self-reported consulting their health care professionals about the depression score provided via the app.

Broken down by country of origin, 26 out of 53 (49%) Australian participants, who followed up, reported consulting their health care professionals regarding their PHQ-9 scores, compared with 23 out of 50 (46%) from Canada, 28 out of 85 (33%) from the United Kingdom, and only 14 out of 51 (27%) from the United States, with no differences between the countries (χ^2^_3_= 7.4, *P*=.059). Eventually, 27 out of 91 (29%) participants self-reported being diagnosed with depression as a result of the consultation with health care professionals.

Logistic regression analysis incorporating demographics (Table 1) and health characteristics variables (Table 3) identified only positive attitude toward depression as a real disease as being associated with increased follow-up response rate OR 3.2, CI 1.38–8.29.

**Table 3:** Health characteristics.

Characteristics		Total n (%)
Alcohol consumption	Never	1218 (48.0)
	1-2 standard drinks/occasion	648 (25.6)
	3-4 standard drinks/occasion	299 (11.8)
	5+ standard drinks/occasion	373 (14.6)
Chronic disease		
	No	1747 (68.8)
	Yes	791 (31.2)
Depression diagnosis		
	No	1934 (76.2)
	Yes	604 (23.8)
High depressive symptoms		
	PHQ-9 cutoff of 11 or above	741 (29.2)
	PHQ-9 less than cutoff of 11	1797 (70.8)
Cigarette smoking		
	No	1725 (67.9)
	Yes, 10 cigarettes or less/day	352 (13.9)
	Yes, 11-20 cigarettes/day	310 (12.2)
	Yes, 21 cigarettes or more/day	151 (5.9)
Attitude toward depression (Depression is a real illness)		
	Strongly agree/Agree	2184 (86.1)
	Neutral	262 (10.3)
	Strongly disagree/Disagree	92 (3.6)
Attitude toward anti-depressant medications (Do they help restore normal level of functioning?)		
	Strongly agree/Agree	1107 (43.6)
	Neutral	1089 (42.9)
	Strongly disagree/Disagree	342 (13.4)
Attitude toward counseling (Help restore normal level of functioning)		
	Strongly agree/Agree	1407 (55.4)
	Neutral	843 (33.2)
	Strongly disagree/Disagree	288 (11.4)

## Discussion

In this study, 2538 participants from Australia, Canada, New Zealand, the United Kingdom, and the United States, completed the PHQ-9 depression screening using a mobile phone app. Of the respondents, 322 participants with high depressive symptoms who had not previously been diagnosed with depression were directed via the app to seek health care professional advice. The app also sent a follow-up message after 1 month using a mobile phone push notification asking users if they had sought health professional advice for the depression score they received from the app. Approximately 74% (239 out of 322) of users who scored highly on the app completed the follow-up, of which 38% (91 out of 239) had self-reported that they had consulted their health care professionals about the depression score provided via the app. The highest proportions of participants who had consulted a health care professional were from Australia and Canada.

### Depression Screening via Mobile Phone App and Motivation to Seek Help

This study demonstrated that mobile phone users from a variety of countries were willing to use the depression-screening app and some acted on the results. More than one-third of the follow-up respondents acted on the recommendation provided via the app and 29% (27 out of 91) self-reported that they had received a diagnosis of depression from their health care professional at follow-up. This shows that mobile phone depression screening can influence users to discuss results obtained from a mobile phone screening test. However, we still do not know how the majority who did not sought help benefited from or used screening results.

The findings of this study on the ability of depression screening to motivate some of those screened to discuss the results with health care professionals are well matched with previous studies [25]. Although in this study we relied on the user to initiate the processes of seeking the health care professional help to interpret the results, such mobile phone mental health screening might be more effective if linked to electronic health records so that clinicians can view it to enhance the communication process. This should also follow the US Preventive Services Task Force’s recent recommendations about screening for depression in the general adult population, which also recommends adequate systems in place to ensure accurate diagnosis, effective treatment, and appropriate follow-up [26].

### Depression Prevalence and Usage of the Depression-Screening App

Different studies have shown a strong relationship between socioeconomic status and the prevalence of depression. In this study, the majority of the participants who used the depression-screening app were in a lower-income demographic and earned less than US $20,000/year [27]. Although the prevalence of depression in the United States is supposedly high and has been reported to affect approximately 1 in 5 people [27,28], there were fewer participants from the United States, 497 (19.6%), than from the United Kingdom, 1108 (43.66%). There was a similar finding, 109 (4.3%) participants in the New Zealand subgroup that had previously been reported to have a high prevalence of depression had a lower response rate in this study. Thus, variations in mobile phone use and app ranking in each country might be the major causes of such variation [29].

### Variation in Seeking Health Care Professional Advice

Australia and Canada had the highest proportions of users 49% (26 out of 53) and 46% (23 out of 50) respectively, who scored high on the app and reported going on to discuss the results with health care professionals. One of the possible reasons why more participants from Australia and Canada sought professional help could have been access to low-cost subsidized government-run health care systems. As previously mentioned, the majority of the participants had incomes of less than $20K/year, 1276 out of 2538 (50.28%). In the United States, the average direct annual per patient costs were $10,402 for bipolar patients and $7494 for depressed patients [30]. Therefore, providing free or affordable health care costs might be a major factor that motivates people with depression to seek professional help after general population screening. In addition, employees in the United States who are treated for depression incur annual per capita health and disability costs of $5415, which is significantly more than other diseases like hypertension, and treatment associated with a mean of 9.86 annual sick days, which is significantly more than other conditions [31].

### Implications

There are various mobile phone apps in app stores that provide mental health or other health screening assessment tools (such as cancer screeners). However, the impact of such apps on consumer decisions is unknown. Moreover, there is no universal app store policy to responsibly direct consumers’ behavior regarding specific therapy [32]. It is recommended that app stores implement a responsible policy to force medical apps to declare that screening results obtained should be discussed with appropriate health care professionals. This criterion should also be included in the quality evaluation of any mobile phone health app. However, while positive screening results via mobile phone apps may stress a user, false negatives may also harm users, demotivating them from seeking health care advice.

Mental health screening apps need to implement functions to better motivate users to act on the provided screening results and encourage them to discuss the results with health care professionals. This study demonstrated that 148 out 239 (62%) respondents who demonstrated significant depressive symptoms did not discuss their results with a health care professional. The screening function alone might therefore not be sufficiently effective in motivating users to seek professional assistance. Improving this utility is critical when designing and implanting mobile phone technology for assistance with health care provision.

In a previous mobile phone depression-screening study, (2642/6089) 43.38% of participants completed the PHQ-9 questionnaire an average 5.3 times in the 4-month study period, with a depression score above 11 at the first test being associated with multiple PHQ-9 completions [3]. In another case study, 13 patients with major depressive disorder used a simple mobile phone app to answer 3 randomly sampled questions from the PHQ-9 survey 3 times per day for the duration of 1 month [33]. Given the feasibility of using mobile phone apps to monitor depressive symptoms, comparing a patient’s current responses to previous responses might help mental health practitioners make informed decisions [34]. Finally, although 2184 out of 2538 (86.05%) participants recorded a positive attitude toward depression as a real disease, only 1107 out of 2538 (43.61 %) had a positive attitude toward antidepressant medications’ ability to restore normal level of functioning. Future mental health mobile phone interventions may need to consider adding a component to improve attitude toward antidepressant medications.

### Limitations

This study focused on simulating the real-world use of such screening tools. It deliberately recruited participants the typical way mobile phone users will seek apps and provided new evidence that relevant users in various countries seek and use mobile phone-based mental health interventions. This process limited the ability to validate the self-reported data, which is less rigorous than clinically validated data. Another limitation of this study is that the passive recruitment strategy might have drawn in participants more motivated to complete the screening and the follow-up, perhaps those aware of their mental health problem. To test the feasibility of using push notifications to follow up with participants, we used a short set of follow-up questions. Therefore, this study did not provide details about the type of depression diagnosis or who provided the depression diagnosis (eg, a general practitioner or specialist). It is also unlikely that the population included in this study is representative of the depression prevalence in the population, as users had to have a mobile phone and be active app users. Moreover, the cohort design lacked a comparison or control group, which can provide results that are more rigorous. Finally, the demographics of users in this study do not reflect the national averages. For example, in the United States, depression rates were higher in 40-59 year olds and women. [35] However, in this study most of the participants were younger and more participants were male than females.

### Conclusion

Previous studies have confirmed the feasibility of depression screening using mobile phone apps in various countries; however, it was unknown if such screening could motivate users to discuss the obtained results with health care professionals, and lead to clinical diagnosis. This study showed that a mobile phone depression-screening app could motivate some users to discuss the obtained results of such tests with health care professionals for further diagnosis and management. However, further study should investigate how other app users use the screening results provided by depression-screening apps.
